# Assessment of non-inferiority with meta-analysis: example of hypofractionated radiation therapy in breast and prostate cancer

**DOI:** 10.1038/s41598-020-72088-2

**Published:** 2020-09-22

**Authors:** Jane-Chloé Trone, Edouard Ollier, Céline Chapelle, Patrick Mismetti, Michel Cucherat, Nicolas Magné, Paul Jacques Zuffrey, Silvy Laporte

**Affiliations:** 1 Département de radiothérapie, Institut de Cancérologie Lucien Neuwirth - Saint Etienne, 42270 St Priest-en-Jarez, France; 2 SAINBIOSE U1059, Equipe DVH, Université Jean Monnet, Saint-Etienne, France; 3 Unité de Recherche Clinique, Innovation, Pharmacologie, Hôpital Nord, CHU de Saint-Etienne, Saint-Etienne, France; 4 Service de Médecine Vasculaire et Thérapeutique, Hôpital Nord, CHU de Saint-Etienne, Saint-Etienne, France; 5 UMR CNRS 5558 Evaluation et Modélisation des Effets Thérapeutiques, Université Claude Bernard Lyon 1, Lyon, France; 6 Département d’Anesthésie-Réanimation, Hôpital Nord, CHU de Saint-Etienne, 42055 Saint-Etienne, France

**Keywords:** Cancer, Oncology

## Abstract

The aim of this study was to propose a methodology for the assessment of non-inferiority with meta-analysis. Assessment of hypofractionated RT in prostate and breast cancers is used as an illustrative example. Non-inferiority assessment of an experimental treatment versus an active comparator should rely on two elements: (1) an estimation of experimental treatment’s effect versus the active comparator based on a meta-analysis of randomized controlled trials and (2) the value of an objective non-inferiority margin. This margin can be defined using the reported effect of active comparator and the percentage of the active comparator’s effect that is desired to be preserved. Non-inferiority can then be assessed by comparing the upper bound of the 95% confidence interval of experimental treatment’s effect to the value of the objective non-inferiority margin. Application to hypofractionated RT in breast cancer showed that hypofractionated whole breast irradiation (HWBI) appeared to be non-inferior to conventionally fractionated RT for local recurrence. This was not the case for accelerated partial breast irradiation (APBI). Concerning overall survival, non-inferiority could not be claimed for either HWBI or APBI. For prostate cancer, the lack of demonstrated significant superiority of conventional RT versus no RT precluded any conclusion regarding non-inferiority of hypofractionated RT.

## Introduction

Except for major therapeutics advances, demonstrate the superiority of a new treatment is a difficult task. Efficacity is often comparable to the standard treatment but new treatment may provide other advantage. For example, in oncology, radiotherapy (RT) is an important component of cancer treatment, approximately 50% of all cancer patients receiving RT during their medical care^1^. More than a third of men with localized prostate cancer are treated with external-beam RT while whole breast irradiation (WBI) remains the standard of care after breast-conserving surgery^2–4^. The duration of treatment of conventionally fractionated radiotherapy is long, 8–9 weeks for prostate cancer and 6–7 weeks for breast cancer. Hypofractionated RT (involving fewer treatments but a higher dose per treatment) has been developed to shorten RT. Hypofractionated RT, has several advantages including convenience for patients, increased treatment capacity based on the α/β model, and decreased cost^5^. Hypofractionated RT is not intended to provide a superiority efficacy than conventionally fractionated RT.

Traditional superiority trials are thus not well suited to evaluate efficacy of such treatments and non-inferiority trials have been proposed. The latter aim to demonstrate that a new treatment is not “inferior”, not “unacceptably worse” to the standard by a too large amount. That amount is called the non-inferiority margin. Demonstration of a non-inferiority requires then a direct comparison between the new treatment and the standard treatment using the pre-defined non-inferiority margin as decision threshold. If non-inferiority of some treatments is clearly demonstrated by high powered and well-designed studies, in most cases available data are not sufficient, and a meta-analysis may be needed to summarize the available evidence. However, the assessment of non-inferiority from the literature faces two issues: (1) the choice of the non-inferiority margin and (2) the non-adapted design of most clinical studies.

First, the choice of the non-inferiority margins is a clinical judgment and may vary greatly between trials. Using a wide non-inferiority margin makes it easier to conclude to the non-inferiority but exposes to the risk to recommend a treatment that is (statistically) inferior to the standard treatment. A recent work has highlighted that non-inferiority margins used in clinical trials were very variable and often did not allow to preserve at least 50% of the active comparator’s effect leading to potentially erroneous conclusions^6^. Summarizing studies’ conclusions obtained with variable non-inferiority margins represent a challenging issue. The calculation of an objectively predefined margin based on the active comparator effect could be a solution.

The second issue is that many studies have been carried out with an inappropriate design. Some of the studies may have been conducted as superiority trials. Even if these studies allow an estimation of the treatment effect, they cannot be individually used for the assessment of non-inferiority as no margin was defined a priori.

The aim of this study was to propose a methodology for the assessment of non-inferiority with meta-analysis. Assessment of hypofractionated RT in prostate and breast cancers was used to illustrate the method. In breast cancer, two modes of hypofractionated RT have been investigated: accelerated partial breast irradiation (APBI) techniques, consisting in a single irradiation limited to the zone in which the risk of recurrence is greatest, and hypofractionated whole breast irradiation (HWBI) where the radiation is given to the whole breast, but in larger daily doses using fewer treatments compared to conventional RT.

## Materials and methods

### Non-inferiority assessment with meta-analysis

To assess the non-inferiority of an experimental treatment versus an active comparator two quantities are needed: i) the value of the non-inferiority margin and ii) an estimation of experimental treatment’s effect versus the active comparator. The procedure proposed in this work is divided in three steps:

The first step is the calculation of an objective non-inferiority margin. As recommended in the FDA guidelines^7^, the non-inferiority margins could be defined on the basis of:

1. the reported effect of active comparator versus control (${HR}_{AC Vs Ctrl}$). If not directly available from the literature, it can be evaluated by performing a meta-analysis of randomized clinical trials. The endpoint used to evaluate this effect needs to be the same as the one used for the evaluation of the experimental treatment effect versus the active comparator in the first step of the procedure.
2. The percentage of the active comparator’s effect that is desired to be preserved.

Based on these two quantities, it is possible to calculate an objective non-inferiority margin that allows to preserve a target percentage of the active comparator’s effect. This calculus can be based either on the mean estimate of the active comparator’s effect (${HR}_{AC Vs Ctrl}$) or on the upper bound of the 95% confidence interval of the estimate of the active comparator’s effect (${UB}_{AC Vs Ctrl}$). The second proposal provides a more conservative margin. Non-inferiority margins can be calculated using the following formulae^6,8^ :

1 $$\delta ={\left(\frac{1}{{HR}_{AC Vs Ctrl}}\right)}^{1-\alpha }and\,{\delta }^{MAX}={\left(\frac{1}{{UB}_{AC Vs Ctrl}}\right)}^{1-\alpha }$$

with $\alpha$ the target percentage of the active comparator’s effect that is desired to be preserved.

The second step is the estimation of the experimental treatment’s effect versus the active comparator based on a meta-analysis of randomized control trials. The endpoint used for the meta-analysis has to be the same as the one used for the evaluation of active comparator’s effect in the first step. In this first step, all the randomized trial that have evaluated the experimental treatment versus the active comparator need to be included. Studies with an inappropriate design, such as superiority trials, should be considered to improve the statistical power of the comparison.

**Figure 1:**
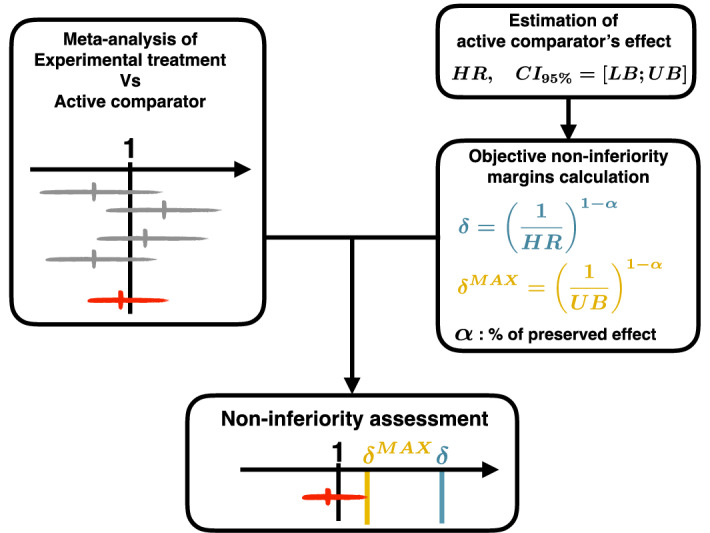
Procedure of non-inferiority assessment using meta-analysis.

Finally, the third step corresponds to the assessment of non-inferiority. It is performed by comparing the upper bound of the 95% confidence interval of the experimental treatment’s effect calculated in step 1 with the objective non-inferiority margins calculated in step 2. The procedure is represented schematically in Fig. 1.

### Application to hypofractionated RT in breast and prostate cancer

#### Definition of non-inferiority margins

For each cancer location and clinical endpoint, the objectives margins were calculated in order to preserve at least 50% of the reported effect of conventionally fractionated RT. We performed a literature search to identify studies or meta-analysis that estimated the effect of conventional fractionated RT for each cancer location.

#### Assessment of hypofractionated RT effect

##### Literature search

We performed a systematic review of published phase 2 and phase 3 randomized clinical trials comparing conventional RT with hypofractionated RT, for prostate or breast cancer. Trials using adjuvant treatments, such as chemotherapy or hormonal therapy, were eligible for inclusion if the adjuvant treatment was used in both the experimental and active control groups.

An independent review of citations in the Medline database from May 1990 (first randomized clinical trial evaluating hypofractionated RT) to July 2019 was conducted. The keywords employed in our search included “clinical trial, hypofractionation, hypofractionated, accelerated radiation therapy, stereotactic and radiosurgery”. The search was not restricted to articles published in English. To verify the completeness of our search, we compared our findings with those of previously published meta-analyses on the same subject. We reviewed every publication identified, and in the event of duplicate publications, the most recent report of the selected clinical trial was included in the meta-analysis.

##### Data collection and analysis—study selection

Two of the authors (JCT and EO) independently evaluated studies for possible inclusion, any disagreements being resolved by discussion.

##### Data extraction and management

Data were independently extracted by two of the authors (JCT and EO). In the event of discrepancies between the reviewers, a consensus was reached by discussion.

For each clinical trial, we extracted details of the study characteristics (names of authors; year of publication; number of patients randomized to each treatment group); study design (double-blind vs. open-label); methodological qualities according to the Cochrane tool, taking into account random sequence generation, concealment of the allocation sequence, blinding of participants and personnel, blinding of outcome assessment, incomplete outcome data and selective reporting (Supplementary Table S1)^9^; patient characteristics (mean age, gender; disease stage, cancer location); characteristics of the radiotherapy regimens evaluated (volume of tissue irradiated, total dose, dose per fraction, methods of radiation); primary outcomes (overall survival (OS), local recurrence in the ipsilateral breast, or biochemical failure (BCF) in prostate cancer), toxicity (including acute and late adverse events in each group) and cosmetic outcome for breast cancer. In the event of missing data, we completed data extraction by reference to previously published meta-analyses^10–13^.

##### Endpoints

The endpoints of our meta-analysis were local recurrence and OS for breast cancer, and BCF and OS for prostate cancer. The hazard ratio (HR) was used to summarize the results for each endpoint. In each individual trial, the HRs for hypofractionated RT compared to conventional fractionated RT and their 95% confidence interval (95% CI) were either extracted from the corresponding publication or derived from the number of deaths in each treatment group and the log-rank *P* value reported in the publication^14^. When the log-rank *P* value was missing, HRs were derived from the survival curves or estimated according to the corresponding risk ratio (RR)^14^.

##### Meta-analyses

We used a fixed-effects model based on the logarithm of the HR weighted by the inverse of the variance to combine the results of individual trials. For each meta-analysis, statistical heterogeneity between the studies was explored using Cochrane’s Q statistic, study consistency being quantified by means of the I^2^ statistic^15^. In the event of significant heterogeneity (*P* value < 0.10) with no clear explanation, a random-effect model^16^ was used for data analysis. To avoid divide-by-zero errors, we used a 0.5 zero-cell correction^17^.

The results of the meta-analyses are presented graphically, including the effect size expressed as the HR with the corresponding 95% CI. An HR equal to 1 indicates no difference between the treatments, an HR less than 1 indicating that hypofractionated RT is better and an HR greater than 1 indicating that the control (conventionally fractionated RT) is better. We explored the publication bias of the studies included in the final analysis using Begg’s funnel plot and Egger’s test (Supplementary Fig. S2)^18,19^.

All statistical analyses were performed using R statistical software, version 3.3.1 with the meta packages (version 4.7).

#### Assessment of historical NI margin

We also extracted the non-inferiority margins used in the literature for each cancer location. Based on Eq. (1), we calculated the percentage $\alpha$ of the effect of conventionally fractionated RT preserved with each of the published non-inferiority margins. This calculation was done using the following formula:

2 $$\alpha =\frac{log\left(\delta \right) + log\left({HR}_{AC Vs Ctrl}\right)}{log\left({HR}_{AC Vs Ctrl}\right)}$$

where $\delta$ corresponds respectively to the non-inferiority margins used in the study of interest and ${HR}_{AC Vs Ctrl}$ to the mean estimate of the active comparator’s effect, the conventionally fractionated RT in this example.

## Results

### Determination of the non-inferiority margins

#### Breast cancer

A previous published meta-analysis of 17 randomized trials in breast cancer explored the efficacy of RT versus no RT^20^. The risk ratio (RR) for local recurrence was 0.50 (95% CI 0.46–0.55) and the RR for OS was 0.92 (95% CI 0.86–0.99) in favor of RT.

To calculate the non-inferiority margins for local recurrence corresponding to 50% of the effect of conventionally fractionated RT, we considered both the value of ${HR}_{CRT Vs Ctrl}$ (0.50) and the value of the upper bound of its 95% CI ${UB}_{CRT Vs Ctrl}$ (0.55).

$${\delta }_{50\%}={\left(\frac{1}{0.50}\right)}^{1-0.5}=1.41\,and\,{\delta }_{50\%}^{MAX}={\left(\frac{1}{0.55}\right)}^{1-0.5}=1.35$$

We also determined objective non-inferiority margins for OS: ${\delta }_{50\%}$= 1.04 and ${\delta }_{50\%}^{MAX}$=1.01 (Table 1).

**Table 1:** Determination of published and objective non-inferiority margins.

	Endpoint	Margin HR			
		Published margins		Objective non-inferiority margins	
		Most permissive	Most conservative	${\delta }_{50\%}$	${\delta }_{50\%}^{MAX}$
HWBI	LR	1.71	1.71	1.41	1.35
	OS	N/A	N/A	1.04	1.01
APBI	LR	2.5	1.42	1.41	1.35
	OS	N/A	N/A	1.04	1.01
Prostate	BCF	1.67	1.19	N/A	N/A
	OS	N/A	N/A	N/A (NS results)	N/A

*HR* hazard ratio; *HWBI* hypofractionated whole breast irradiation, *APBI* accelerated partial breast irradiation, *N/A* not available; *NS* non-significative; *BCF* biochemical failure; *LR* local recurrence; *OS* overall survival; ${\delta }_{50\%}$*and* ${\delta }_{50\%}^{MAX}$: non-inferiority margins corresponding to 50% of the effect of conventionally fractionated RT, according to the value of ${HR}_{CRT Vs Ctrl}$ and the value of its 95% CI upper bound, respectively.

#### Prostate cancer

With regard to historical trials evaluating the efficacity of conventional fractionated RT in prostate cancer, our literature search identified only one study, which compared conventional fractionated RT with surveillance^21^. As regards OS, the hazard-ratio was non-significant (0.51 [95% CI, 0.15–1.69]). No data concerning BCF were reported. The upper limit of the 95% CI for OS was above 1 (1.69) and did not permit the calculation of ${\delta }_{50\%}$.

### Experimental treatment’s effect versus active comparator

#### Identification and description of relevant studies

**Figure 2:**
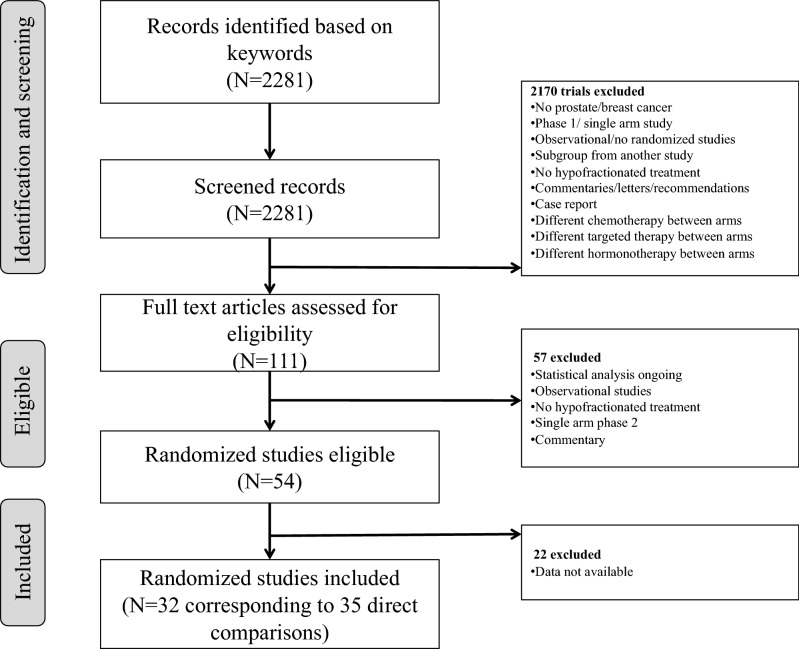
Flow chart of trial selection.

Our search procedure identified 2,281 potentially relevant clinical trials. After a review of the publications, 32 trials were considered eligible for inclusion in the meta-analysis, yielding a total of 19,623 patients and 35 direct comparisons (3 studies having multiple arms) (Fig. 2).

The baseline characteristics of the studies included are presented in supplementary (Table S3). Twenty studies concerned breast cancer and 12 studies concerned prostate cancer^22–33^. Among the 7 APBI^34–40^ studies, 6 were designed as non-inferiority trials and one did not specify the design of the trial. In the 13 HWBI^41–53^, 2 were designed as non-inferiority trials, 2 as superiority trials andin 9 studies did mention the design of the trial. In the 12 prostate cancer studies, 5 were designed as non-inferiority trials, 4 as superiority trials and 3 did not report the design of the trial Among the 13 studies designed as non-inferiority trials, the non-inferiority margin was specified in 9 cases (69%). The primary endpoint was toxicity in 15 trials, local recurrence in 10 trials, and BCF-free survival in 5 studies.

#### Hypofractionated whole breast irradiation

Local recurrences and OS were documented in 9 and 5 of the 13 HWBI studies respectively.

**Figure 3:**
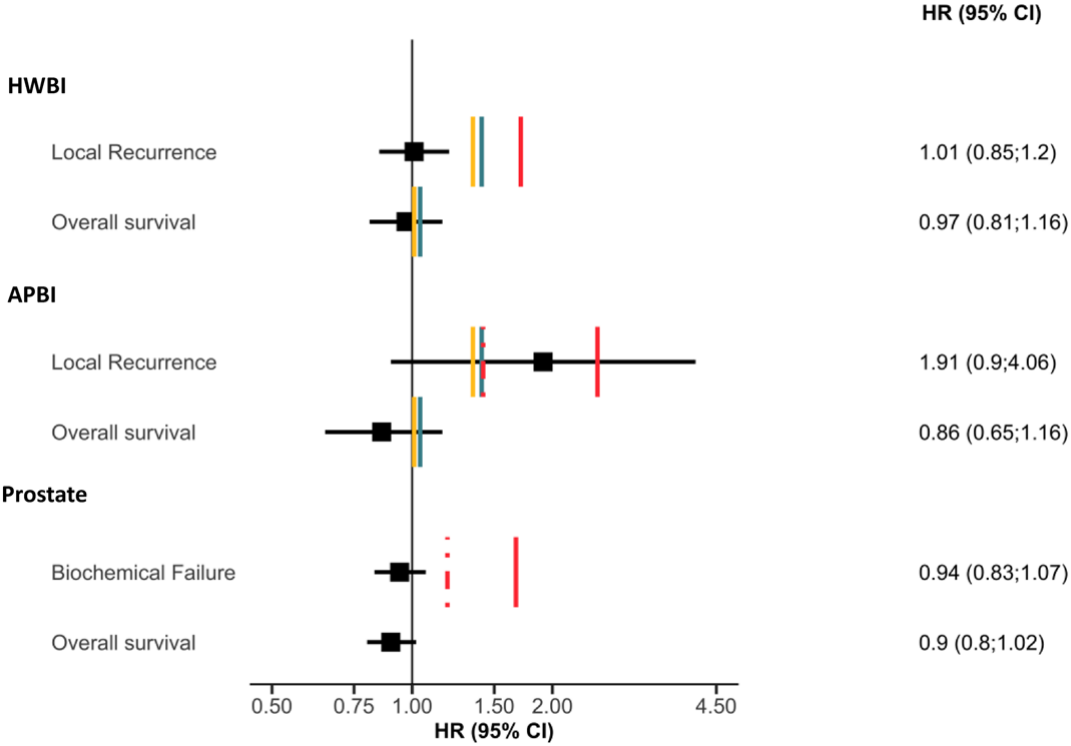
Forest plot representing the estimates of hypofractionation efficacy obtain with meta-analysis compared to the calculated objective margins, most permissive and most conservative published margin for each cancer location and clinical endpoint. *HR* hazard ratio; *CI* confidence interval; *HWBI* hypofractionated whole breast irradiation, *APBI* accelerated partial breast irradiation. Red solid lines: most permissive published margin; red dashed lines: most conservative published margin; blue lines: objective non-inferiority margins corresponding to 50% of the effect of conventionally fractionated RT according to the value of ${HR}_{CRT Vs Ctrl}$ (${\delta }_{50\%}$); yellow lines: objective non-inferiority margins corresponding to 50% of the effect of conventionally fractionated RT according to the value of the 95% CI upper bound of ${HR}_{CRT Vs Ctrl}$ (${\delta }_{50\%}^{MAX}$).

Figure 3 represents for each clinical endpoint the estimates of hypofractionation efficacy obtain with meta-analysis compared to the calculated objective margins, most permissive and most conservative published margin.

For local recurrence, whatever the margin considered, hypofractionation seemed to be non-inferior to conventional-fractionated group (HR 1.01, 95% CI 0.85–1.20).

Objectively calculated non-inferiority margins were very close to 1 (1.01 et 1.04) and did not allow any conclusion to be drawn regarding the non-inferiority of hypofractionated RT (HR 0.97, 95% CI 0.81–1.16). Forest plots of the meta-analysis are displayed in the supplementary data (Supplementary Fig. S4).

#### Accelerated partial breast irradiation

Local recurrences were documented in 6 of the 7 articles identified concerning APBI and OS in 5 of these studies.

Whatever the margins considered, we could not demonstrate the non-inferiority of hypofractionated RT as regards to recurrence (HR 1.91, 95% CI 0.90–4.06) (Fig. 3). With respect to OS, as in the case of HWBI, it was not possible to draw any conclusion as the objectively calculated margins were close to 1 (HR 0.86, 95% CI 0.65–1.16). Forest plots of the meta-analysis are displayed in the supplementary data (Supplementary Fig. S5).

#### Prostate cancer

BCF and OS were documented in 10 of the 12 prostate cancer studies. Published margins were only available for BCF.

Non-inferiority of hypofractionation on BCF is only suggested on the basis of the reported margin (HR 0.94, 95% CI 0.83–1.07) (Fig. 3). For OS, no conclusion could be drawn in the absence of any published or calculable margin (HR 0.90, 95% CI 0.80–1.02). Forest plots of the meta-analysis are displayed in the supplementary data (Supplementary Fig. S6).

### Assessment of historical NI margin

We extracted from the studies included in the meta-analysis the non-inferiority margins used within each non-inferiority trial (See Table 1 and Supplementary Table S3) and then calculated the percentage of the effect of conventionally fractionated RT preserved with each of the published non-inferiority margins using Eq. (2).

**Figure 4:**
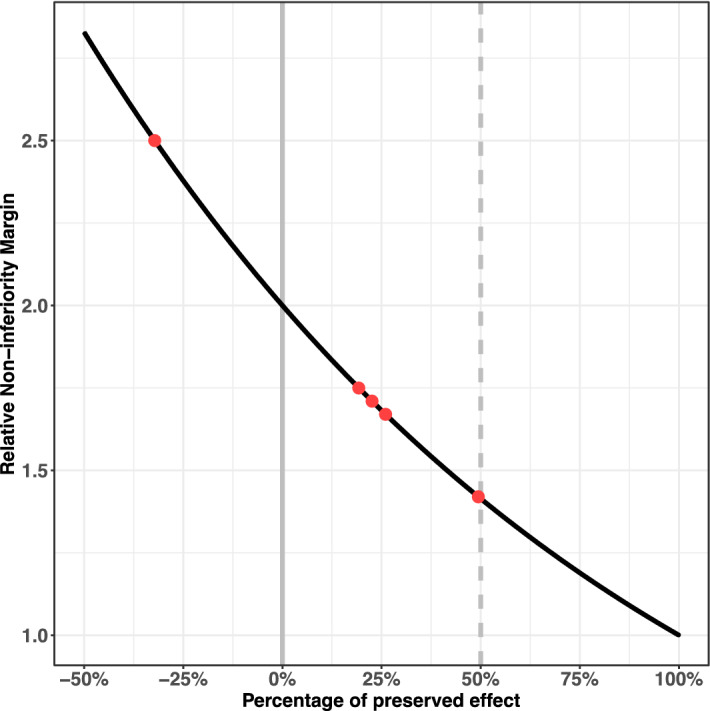
Relation between the percentage of preserved effect of conventional RT and the corresponding relative non-inferiority margin in breast cancer. Red points highlight the percentage of preserved effect of conventional RT calculated for each non inferiority margins used in the literature.

With regard to HWBI and APBI, Fig. 4 represents the relationship between the value of the non-inferiority margin and the percentage of preserved effect of conventionally fractionated RT for local recurrence. Only one study used a non-inferiority margin corresponding to an effect close to 50% of the conventionally fractionated RT’s effect^38^. Other studies only allowed to retain 25% of the effect of conventional RT^34,37,42^. One study have a negative percentage of preserved effect and so could lead to accept treatment with a deleterious effect^39^.

For OS, no published margin was available. For APBI, the most conservative margin used in the literature was close to objective margin (1.42 vs 1.41).

Concerning prostate cancer this calculation was impossible as no randomized study in prostate cancer demonstrated a significant superiority of conventional RT versus no RT.

## Discussion

The present work proposed to assess the non-inferiority of an experimental treatment compared to an active comparator using a meta-analytical approach. The proposed method is based on a two steps procedure. The first step corresponds to an estimation of experimental treatment’s effect versus the active comparator while the second step corresponds to the calculation of an objective non-inferiority margin. The method is illustrated using the comparison between hypofractionated RT and conventional RT for breast and prostate cancer. These two examples highlight the different issues that can occur when performing such analysis.

Controlled trials with a non-inferiority design have the advantage of potentially providing proof that the difference between a novel treatment and an active control treatment is sufficiently small to support authorization of the proposed new treatment. Non-inferiority trials are particularly appropriate in the case of hypofractionated RT, a mode of treatment with clear practical and financial advantages, to verify the absence of loss of efficacity in comparison to conventional RT. However, there are rules concerning appropriate use of the non-inferiority study design to provide evidence of comparative efficacy^7^. First, the effectiveness of the standard treatment needs to be demonstrated. Second, the non-inferiority margins employed should be pre-specified and preferably justified on clinical grounds^54,55^.

An excellent demonstration of the non-inferiority of HWBI was obtained for local recurrence. First, conventional RT resulted in a reduced risk of local recurrence in comparison to no treatment. Second, irrespective of the non-inferiority margin used, HWBI appeared to be non-inferior to conventional RT. With regard to OS, no conclusion could be drawn because the objectively calculated margins were very close to 1.

Concerning APBI, we could not conclude that hypofractionated APBI was non-inferior to conventional RT in the light of the prespecified margins and their 95% confidence intervals.

In prostate cancer, we found no evidence proving the efficacy of conventional RT compared to no RT. This lack of data makes it impossible to define an objective non-inferiority margin.

Concerning the prespecified margins reported, most of the published trials included in our meta-analysis used a non-inferiority margin that was too wide to ensure preservation of a sufficient proportion (50%) of the active comparator’s effectiveness, probably in order to more easily conclude non-inferiority of hypofractionated RT. Determination of the margin in a non-inferiority trial should be based on both statistical reasoning and clinical judgement, but most of the studies offered no justification for their prespecified margins. For example, the use of conventional RT as the active comparator in studies concerning prostate cancer, when this treatment has not been shown to have significant efficacy in previous randomized trials, complicates the definition of a non-inferiority margin. At the same time, the use of a placebo as the comparator does not appear to be ethical in cancer patients. Despite the increased interest in methodology and the definition of non-inferiority margins during recent years, more efforts are required to avoid erroneous declarations of non-inferiority^56^. A consensus on the preferred method for defining non-inferiority margins is needed.

Our study had several limitations. First, the relevance of intention-to-treat analysis versus per-protocol analysis was not addressed. Per-protocol analysis, by excluding patients who failed to complete the full course of treatment, is more likely to reflect differences between the two treatments compared. Second, the objective of the studies included in the meta-analysis (non-inferiority versus superiority) was in general not clearly defined and so no non-inferiority margins were specified. Finally, the cutoff used to define adequate preservation of the active comparator’s treatment effect (50%) was subjective^6^ and in some settings may be clinically insufficient.

In conclusion, we found that hypofractionated RT seemed to be non-inferior to conventional RT only in the context of HWBI and with respect to the endpoint of local recurrence. APBI was not as effective as WBI following our methodology. No conclusion could be drawn for prostate cancer, as the use of an active comparator (conventional RT) in this context did not significantly improve overall survival and the efficacy of conventional RT with regard to BCF has not been evaluated in any clinical study.

It is meaningless to declare non-inferiority of a novel intervention in the absence of any prior evidence of the effectiveness the active comparator. It would be of interest to compare the use of hypofractionated RT with active monitoring in prostate cancer.

## Supplementary information

Supplementary Information.

## Data Availability

The datasets analyzed during the current study are available from the corresponding author on request.
