# Supplementary material for: Assessment of non-inferiority with meta-analysis: example of hypofractionated radiation therapy in breast and prostate cancer
Source: Sci Rep. 2020 Sep 22;10:15415. doi: 10.1038/s41598-020-72088-2 (PMC7508968; doi:10.1038/s41598-020-72088-2)
Supplement: Supplementary file 1 — Supplementary Information. [file 41598_2020_72088_MOESM1_ESM.docx]

**Assessment of non-inferiority with meta-analysis: example of hypofractionated radiation therapy in breast and prostate cancer**

**Supplementary material**

Jane-Chloé Trone1,2*, Edouard Ollier2,3, Céline Chapelle2,3, Patrick Mismetti2,3,4, Michel Cucherat5, Nicolas Magné1, Paul Jacques Zuffrey^2,3,6^, Silvy Laporte2,3,4

*^1^Département de radiothérapie, Institut de Cancérologie Lucien Neuwirth - Saint Etienne (France)*

*^2^SAINBIOSE U1059, Equipe DVH, Université Jean Monnet, Saint-Etienne, France*

*^3^Unité de Recherche Clinique, Innovation, Pharmacologie, Hôpital Nord, CHU de Saint-Etienne, France*

*^4^Service de Médecine Vasculaire et Thérapeutique, Hôpital Nord, CHU de Saint-Etienne, France*

*^5^UMR CNRS 5558 Evaluation et Modélisation des Effets Thérapeutiques, Université Claude Bernard Lyon 1, Lyon, France*

*^6^Département d’Anesthésie-Réanimation, Hôpital Nord, CHU de Saint-Etienne, F-42055, Saint-Etienne, France*

| Author, year | Random sequence generation | Allocation concealment | Blinding of participants and personnel | Blinding of outcome assessment | Incomplete outcome data | Selective reporting |
| --- | --- | --- | --- | --- | --- | --- |
| Spooner, 2012 | ? | ? | - | - | + | + |
| Whelan, 2010 | + | + | - | - | + | + |
| Owen, 2006 | + | + | - | - | + | - |
| Fragkandrea, 2013 | ? | ? | - | - | ? | - |
| Li, 2014 | ? | ? | - | - | ? | + |
| Saha, 2009 | ? | ? | - | - | ? | + |
| Patni, 2012 | ? | ? | - | - | ? | - |
| Haislund, 2012 | ? | ? | - | - | ? | - |
| Haislund, 2012 | ? | ? | - | - | ? | - |
| Agrawal, 2011 | ? | ? | - | - | + | - |
| Van Parijs, 2012 | ? | ? | - | - | ? | - |
| Taher, 2005 | ? | ? | - | - | + | + |
| Baillet, 1990 | ? | ? | - | - | ? |  |
| Barsoum, 2010 | ? | ? | - | - | + | + |
| Livi, 2015 | ? | ? | - | - | + | + |
| Rodriguez, 2013 | ? | ? | - | - | + | - |
| Strnad, 2016 | + | + | - | - | + | - |
| Vaidya, 2010 | + | + | - | - | + | + |
| Veronesi, 2013 | + | + | - | - | + | + |
| Struikmans, 2016 | ? | ? | - | - | + | - |
| Catton, 2017 | + | + | - | - | + | + |
| Dearnaley, 2016 | + | + | - | - | + | + |
| Lee, 2016 | + | + | - | - | + | + |
| Lukka, 2005 | + | + | - | - | + | + |
| Arcangeli, 2017 | ? | ? | - | - | + | + |
| Yeoh, 2011 | ? | ? | - | - | + | + |
| Pollack, 2013 | + | + | - | - | + | + |
| Incrocci, 2016 | + | + | - | - | + | + |
| Hoffman, 2014 | ? | ? | - | - | + | + |
| Norkus, 2009 | ? | ? | - | - | + | + |
| Marzi, 2009 | ? | ? | - | - | ? | + |
| Hoffman, 2018 | + | + | - | - | + | + |

**Supplementary Table S1** Assessment of the risk of bias according to the Cochrane Collaboration’s tool

**Supplementary Figure S2** Characteristics of the studies included and published non-inferiority margins

HWBI: hypofractionated whole breast irradiation, APBI: accelerated partial breast irradiation; LR: local recurrence; BCF: biochemical failure; OS: overall survival

| Radiation location | Author | Type | Primary endpoint | NI margin of the study |
| --- | --- | --- | --- | --- |
| HWBI | Spooner | Superiority | Local recurrence | N/A |
|  | Whelan | NI | Local recurrence | 1.71*† |
|  | Owen (50 Vs 42.9 and 39 Gy) | NI | Toxicity | N/A |
|  | Fragkandrea | N/A | Toxicity | N/A |
|  | Li | N/A | Local recurrence | N/A |
|  | Saha | N/A | Local recurrence | N/A |
|  | Patni | N/A | Toxicity | N/A |
|  | Haislund | N/A | Toxicity | N/A |
|  | Agrawal (50 Vs 30 and 28.5 Gy) | N/A | Toxicity | N/A |
|  | Van Parijs | Superiority | Toxicity | NA |
|  | Taher | N/A | Toxicity | N/A |
|  | Baillet | N/A | Toxicity | N/A |
|  | Barsoum | N/A | Toxicity | N/A |
| APBI | Livi | NI | Local recurrence | 1.67 |
|  | Polgar | NI | Local recurrence | N/A |
|  | Rodriguez | NI | Local recurrence | N/A |
|  | Strnad | NI | Local recurrence | 1.75 |
|  | Vaidya | NI | Local recurrence | 1.42* |
|  | Veronesi | NI | Local recurrence | 2.5† |
|  | Struikmans | N/A | Toxicity | N/A |
| Prostate | Catton | NI | BCF | 1.32 |
|  | Dearnaley (74 Vs 57 and 60 Gy) | NI | BCF | 1.21 |
|  | Lee | NI | DFS | 1.67† |
|  | Lukka | NI | BCF | 1.19* |
|  | Arcangeli | Superiority | Toxicity | N/A |
|  | Yeoh | Superiority | Toxicity | N/A |
|  | Pollack | Superiority | BCF | N/A |
|  | Incrocci | NI | Toxicity | N/A |
|  | Hoffman | N/A | Toxicity | N/A |
|  | Norkus | N/A | Multiple endpoints | N/A |
|  | Marzi | N/A | Toxicity | N/A |
|  | Hoffman | Superiority | BCF | N/A |

**Supplementary Table S3** Characteristics of the studies included and published non-inferiority margins

* most conservative non-inferiority margin; † most permissive margin; HWBI: hypofractionated whole breast irradiation, APBI: accelerated partial breast irradiation, Gy: Gray; NI: non-inferiority; N/A: not available; BCF: biochemical failure; DFS: disease-free survival; VS: versus.

**Supplementary Figure S4** Forest Plots of meta-analysis of randomized controlled trials testing hypofractionated whole breast irradiation on breast cancer (A: local recurrence; B: overall survival). The size of the symbols is proportional to the number of included patients.

**Supplementary Figure S5** Forest Plots of meta-analysis of randomized controlled trials testing accelerated partial breast irradiation on breast cancer (A: local recurrence; B: overall survival). The size of the symbols is proportional to the number of included patients.

**Supplementary Figure S6** Forest Plots of meta-analysis of randomized controlled trials testing hypofractionation on prostate cancer (A: biochemical failure; B: overall survival). The size of the symbols is proportional to the number of included patients.
